# Altered expression of long non-coding RNA GAS5 in digestive tumors

**DOI:** 10.1042/BSR20180789

**Published:** 2019-01-18

**Authors:** Shounan Lu, Zhilei Su, Wen Fu, Zhankun Cui, Xingming Jiang, Sheng Tai

**Affiliations:** Department of Hepatopancreatobiliary Surgery, The Second Affiliated Hospital of Harbin Medical University, Harbin 150086, China

**Keywords:** digestive system tumor, GSA5, large intervening non-coding RNA, single nucleotide polymorphisms

## Abstract

Cancer has become one of the most important diseases that affect human health and life. The effects of cancer in the digestive system are particularly prominent. Recently, long non-coding RNA (lncRNA) has attracted the attention of more and more researchers and has become an emerging field of gene research. The lncRNA growth arrest-specific 5 (GAS5) is a novel lncRNA that has attracted the attention of researchers in recent years and plays an important role in the development of tumors, especially in digestive system tumors. GAS5 was first identified in a mouse cDNA library. It was generally considered that it has the role of tumor suppressor genes, but there are still studies that have a certain ability to promote cancer. Furthermore, the 5-bp indel polymorphism (rs145204276) in the GAS5 promoter region also has a carcinogenic effect. The discovery of GAS5 and in-depth study of single nucleotide polymorphism (SNP) mechanism can provide a new way for the prevention and treatment of digestive system tumors.

## Introduction

In humans, over 70% of genome is continuously transcribed. In fact, only 1–2% of the genome is coding genes, while 80–90% non-coding regulatory elements are transcribed into long non-coding RNAs (lncRNAs) [1]. LncRNAs belong to a family of transcripts longer than 200 nts without or with low protein-coding potential. In human genome, 15787 lncRNA transcripts from 14470 lncRNA genes have been identified [2]. LncRNAs are polyadenylated and catalyzed by RNA polymerase II, and can perform various biological functions in nuclei and cytoplasm [3]. Although lncRNAs have once been thought as the ‘dark matter’ of the genome, an abnormal expression of lncRNAs virtually participates in all stages of cancer development, including cancer initiation, progression, and metastasis.

Single nucleotide polymorphisms (SNPs) are single base pair positions in genomic DNA at which alternative alleles occur [4]. SNPs are the predominant forms of sequence variations in both plant and animal genomes, and it is reported that approximately 150 million SNPs have been discovered in the human genome [5,6]. SNPs can be divided into transversions (C/G, A/T, C/A, and T/G) and transitions (C/T or G/A). The majority of SNPs at any given site are bi-allelic, but tri-allelic and tetra-allelic SNPs also exist [4]. SNPs can be synonymous or non-synonymous. The former do not cause a change in the amino acid being translated, but the latter result in a different amino acid being translated. Non-synonymous SNPs within a transcribed gene can alter its protein structure or function, thereby affecting an organism’s development or response to environment [7]. SNPs’ interactions also play an important role in the development of the disease, especially in cancer research, such as breast cancer and colorectal cancer [8,9].

In recent years, the number of patients suffering from digestive system cancer has gradually increased, and the search for relevant factors for the treatment of digestive tract tumors is imminent. LncRNA growth arrest-specific 5 (LncRNA GAS5) is a new type of lncRNA that has attracted researchers’ attention recently and plays an important role in the development of tumors, especially in digestive system tumors. This article summarizes the expression and mechanism of GAS5 in digestive system tumors.

### LncRNA GAS5

GAS5 is a growth suppressor that is up-regulated when cell growth inhibition is caused by starvation or rapamycin in T cells [10]. The GAS5 gene, which was first reported by Coccia et al. (1992) [43], was isolated from mouse genomic DNA and structurally characterized [11]. The gene is located at 1q25, a total of 630 nts, 12 exons and contains only a short ORF and is considered to have no function to encode proteins; rather these exons are spliced to yield two possible mature lncRNAs, termed GAS5a and GAS5b, due to the presence of alternative 5′-splice donor sites in exon 7 [12]. The abundance of GAS5 transcripts goes up upon cell growth arrest which induced by serum starvation or treatment with rapamycin [13]. It is explained by the interplay between the mammalian target of rapamycin (mTOR) pathway and the nonsense-mediated decay (NMD) pathway [13,14]. Many studies have shown that GAS5 is mainly used as a tumor suppressor involved in cell proliferation, apoptosis, cell migration and invasion [11]. The exon 12-derived stem-loop region of lncRNA GAS5 was found to mimic the glucocorticoid receptor response element (GRE) structurally, and lncRNA GAS5 was shown to compete with GRE to associate with the DNA-binding domain of glucocorticoid receptor. Additionally, lncRNA GAS5 acts as decoy of GRE to inhibit the downstream gene expression of GRE such as cellular inhibitor of apoptosis 2 (cIAP2) and triggers apoptosis during starvation [15]. As research continues to deepen, scholars have discovered that GAS5 can regulate the expression of genes and cellular signals which participate in cell cycle regulation and cellar metabolism by interacting with miRNAs, such as miR-103 and miR-222 have been identified as downstream targets of GAS5 [16,17].

### GAS5 and digestive system tumors

#### GAS5 in hepatocarcinoma

Hepatocarcinoma is one of the most common tumors in digestive system tumors, and GAS5 plays an important role in hepatic carcinoma. GAS5 expression was significantly down-regulated in clinical pathology specimens, and there was a significant negative correlation with tumor size, lymph node metastasis, and clinical stage. However, there was no significant correlation between the level of GAS5 expression and the number of tumors, the presence or absence of HBsAg, and the level of AFP [18]. Further research shows that overexpression of GAS5 can inhibit the invasive ability of hepatocarcinoma cells, mainly because the epithelial–mesenchymal transition (EMT) process is affected [19]. EMT is not only a mechanism for forming fibroblast-like cells but also a process that leads to the migration, invasion, and metastasis of cancer cells. EMT has been shown to be very important in the early events of metastatic spread of tumor cells, which can make cells more active and invasive [20]. Immunohistochemistry showed that Vimentin (Vim) protein was significantly up-regulated in hepatocarcinoma cells, while E-cadherin (E-cad) was down-regulated in tumor tissues. *In vitro*, overexpression of GAS5 can reduce Vim protein significantly, and GAS5 will increase the expression of E-cad, indicating that GAS5 regulates the proliferation and invasion of hepatoma cells by regulating Vim [19]. SNP also plays an important role in the development of cancer. It was found that there is also a link between the 5-bp indel polymorphism (rs145204276) in the GAS5 promoter region and hepatocarcinoma. Interestingly, the opposite of rs145204276 and GAS5, rs145204276 can increase the susceptibility of hepatocarcinoma [21]. Li et al. found that indel polymorphism rs145204276 may influence GAS5 transcriptional activity by affecting the methylation status of a CpG island in the promoter region of GAS5, thereby affecting its utility in suppressing tumors [21]. However, this tumor-promoting mechanism still needs further study, and how to determine the existence of indel polymorphism rs145204276 before treatment is a problem we need to further study. The mechanism of action of GAS5 in liver cancer cells is not well understood, and even the target genes that are directly acting are not found, which also needs to be studied.

#### GAS5 in colorectal cancer

GAS5 also plays a role in regulating tumor growth in colorectal cancer. The study found that GAS5 expression was significantly reduced in colorectal cancer tissue samples compared with adjacent healthy tissues [22]. The low GAS5 expression group showed a significantly increased tumor size [22]. In addition, patients with low levels of *GAS5* mRNA have a shorter survival time, and statistical studies have found that the expression level of GAS5 can be an independent risk factor for colorectal cancer and a predictor of prognosis [22]. Cell experiments revealed that overexpression of GAS5 can significantly inhibit the proliferation rate of colorectal cancer cells, inhibit colorectal cancer cell growth and colony formation, and induce cell cycle G_0_/G_1_ arrest and apoptosis [22]. Further studies showed that the relative expression levels of *casp9* mRNA and pho-Casp9 protein were increased in GAS5-expressing tissues, and Akt, extracellular regulated protein kinases (ERK), *Casp3* mRNA, p-Akt, p-ERK, and pho-Casp3 proteins were decreased [23]. In addition, some scholars found that miR-221 and miR-182-5p are highly expressed in colorectal cancer cells and have a significant negative correlation with GAS5. Further studies by Wang et al. showed that overexpression of GAS5 can inhibit the expression of miR-221 and miR-182-5p, thereby reducing the proliferation, migration, and invasion of colorectal cancer cells, but the specific mechanism needs further analysis [24,25]. Zheng et al. [11] found that rs145204276 also had the ability to enhance colorectal cancer susceptibility and promote lymph node metastasis of tumor in colorectal cancer tissues. This is consistent with the results of Li et al. [23], but it is not known whether the mechanism of action is the same or not. GAS5 has a relatively large number of studies in colorectal cancer, but unfortunately the research is not deep enough, and no clear regulation of gene expression axis has been found. I think this is the direction the researchers are working on next.

#### GAS5 in pancreatic cancer

Pancreatic cancer is one of the malignant tumors of the digestive tract. It has a high degree of malignancy and is difficult to diagnose and treat. The 5-year survival rate is only 6% [26,27]. *In vitro*, GAS5 was found to inhibit the viability, migration, and invasion of pancreatic cancer cells. After transfection of pcDNA GAS5, the cell viability of the tumor cells was significantly reduced, the percentage of apoptosis was significantly increased, and the tumor weight was significantly reduced [28]. In human pancreatic cancer tissues, Gao et al. [28] found a significant decrease in the expression of GAS5 and phosphatase and tensin homolog (PTEN) and a significant increase in miR-32-5p. Further experiments showed that GAS5 can promote the expression of *PTEN* mRNA, but miR-32-5p can increase the level of *PTEN* mRNA. And then, PTEN blocks the activation of PI3K/Akt signaling pathway and inhibits pancreatic cancer cell proliferation and survival. This indicates that miR-32-5p negatively regulates the expression of PTEN and mediates the effect of GAS5 on the expression of PTEN, which in turn affects pancreatic cancer proliferation [28]. In addition, scholars have found that GAS5 not only inhibits the proliferation of PC cells, but also has an impact on drug resistance of drug-resistant cells in recent years. In drug-resistant tumor cells, *GAS5* mRNA levels were significantly decreased, and miR-181c-5p expression was increased in drug-resistant cells, and there was a negative correlation between them. Up-regulation of GAS5 can increase the expression of mammalian sterile 20-like kinase 1 (MST1) protein and promote phosphorylation of yes-associated protein (YAP) and transcriptional co-activator with PDZ-binding motif (TAZ), whereas overexpression of miR-181c-5p can reverse this effect [29]. It has been confirmed in previous literature that miR-181c-5p is a key repressor of Hippo signaling by targetting the core kinase box, namely MST1, while Hippo signaling pathway inactivation and YAP/TAZ overactivation play an important role in chemotherapeutic drug resistance. This shows that Hippo signal may be a new target for cancer chemotherapy, and GAS5 can inhibit the effect of miR-181c-5p on drug resistance [30,31]. It has also been reported in the literature that GAS5 acts as a competitive endogenous RNA with miR-221 to inhibit PC cell growth, metastasis, and gemcitabine resistance [32]. This provides a new way to solve the problem of pancreatic cancer chemotherapy resistance. In addition, there are reports that GAS5 acts as a tumor suppressor by modulating the expression of the oncogene cyclin-dependent kinase 6, but the specific regulatory mechanisms have not been elaborated, and further studies are needed to confirm [10].

#### GAS5 in gastric cancer

Gastric cancer is one of the most aggressive malignant tumors. Despite significant advances in molecular biology research of gastric cancer, effective strategies to reduce the incidence and mortality of gastric cancer are still insufficient. The researchers said that GAS5 plays an important role in inhibiting the growth of gastric cancer and that knockdown of GAS5 will eliminate the cell cycle arrest of tumor cells. Meanwhile, the researchers found that as the GAS5 knockdown Y-box binding protein 1 (YBX1) protein levels are gradually reduced [10]. YBX1 is a DNA/RNA binding protein that regulates the level of p21 protein in cells [33]. As we all know, p21 protein is a G_1_ phase regulator. After the decrease in YBX1 expression, the expression of p21 also decreases, and the percentage of cells in G_1_ phase decreased [10]. This indicates that down-regulation of GAS5 expression accelerates the depletion of YBX1 protein and decreases the expression of p21 protein, thereby eliminating G_1_ arrest. In conclusion, the GAS5/YBX1/p21 pathway controls the proliferation of gastric cancer cells [10]. Interestingly, Han et al. found that GAS5 also plays a role in improving gastric cancer sensitivity to chemotherapy [34]. GAS5 and miR-23a were negatively expressed in gastric cancer cells, and overexpression of GAS5 significantly reduced miR-23a expression. Further studies revealed that metallothionein 2A (MT2A) also has a high expression level in GAS5-overexpressing cells, whereas after transfection of miR-23a, MT2A decreased. This demonstrates that the GAS5/miR-23a/MT2A pathway does exist. Moreover, studies have shown that MT2A is the main component of the gastrointestinal mucosal barrier, and elevated MT2As can increase the chemosensitivity of tumors through the NF-κB pathway [35,36]. This means that GAS5 can increase the sensitivity of gastric cancer chemotherapy by modulating miR-23a and regulating MT2A expression [34]. In addition, some scholars have studied the role of GAS5 gene variant rs145204276 in gastric cancer cells. Li et al. [37] found high expression of rs145204276 in gastric cancer cells, but he did not think that this is an expression that promotes gastric cancer cell proliferation but a protective mechanism of the body in gastric cancer cells. However, its mechanism has not been studied in detail, so the mechanism of high expression of rs145204276 in gastric cancer cells is still unknown [37]. The discovery that GAS5 can improve the chemosensitivity of gastric cancer is considered to be very important and has good clinical application value, which deserves special attention.

#### GAS5 in esophageal carcinoma

Esophageal carcinoma is one of the digestive system malignancies. However, the role of GAS5 in esophageal cancer has two completely different views. Bai et al. studies suggest that GAS5 plays a tumor suppressive role in esophageal cancer tissues [38]. In animal experiments, the growth curve and average weight of tumors of mice injected with the GAS5 siRNA group were significantly larger than those of the control group. And GAS5 expression in stage III and IV esophageal cancer tissue was significantly lower than that of stage I and II esophageal cancer tissue. In contrast, the expression of miR-196a in stage I and II esophageal squamous cell carcinoma tissues was lower than that in III and IV tissues [38]. The study found that GAS5 expression was down-regulated in esophageal cancer cell lines, while miR-196a was up-regulated, and GAS5 and miR-196a were negatively correlated in esophageal cancer cells. *In vitro*, it is found that miR-196a binds to exons via RISC to regulate the expression of GAS5 so as to inhibit tumors [38]. However, Lu et al.’s study believes that RAS5 plays a role in promoting tumor growth in tumor cells [39]. The major regulatory mechanisms are the GAS5/miR-301a/CXCR4/Wnt/β-catenin (β-cat) and NF-κB signaling pathway regulatory networks. GAS5 acts as an endogenous sponge to inhibit miR-301a expression, whereas a decrease in miR-301a expression promotes the expression of CXCR4. Western blot results showed that down-regulation of B-cell lymphoma-2 (Bcl-2), up-regulation of Bax, and activation of cleaved-caspase-3 and cleaved-caspase-9 expression were associated with CXCR4 overexpression and low expression of miR-301a [39]. Further *in vitro* experiments found that overexpression of CXCR4 reduced the inhibitory effect of Wnt/β-cat and NF-κB signaling pathway on tumor proliferation [39]. Although the effect of GAS5 is completely different in the two papers, the important role of GAS5 in esophageal cancer is undeniable. The results of these two studies on GAS5 in esophageal cancer are contradictory. We need to further explore its role in esophageal cancer and the causes of two different outcomes, which will help in the treatment of esophageal cancer in the future.

## Conclusion and prospects

**Figure 1:**
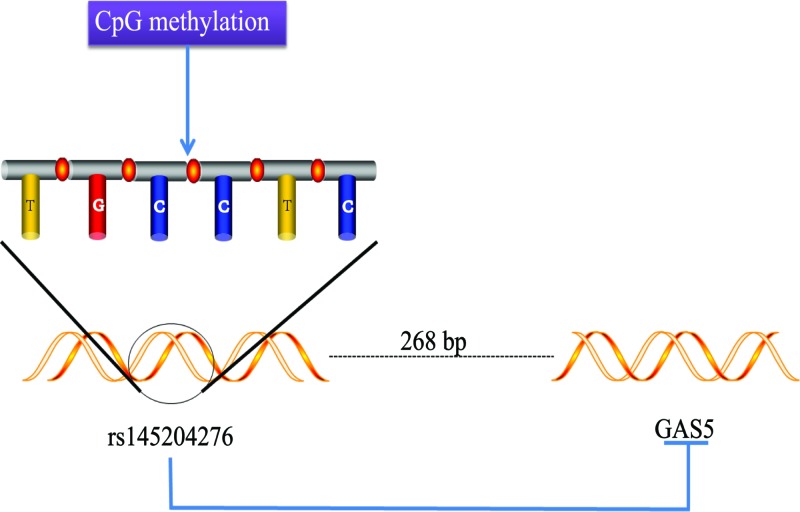
Influence of rs145204276 on GAS5 rs145204276 is located 268-bp upstream of the GAS5 promoter, and CpG methylation acts on its TGCCTC site to inhibit the tumor suppressive effect of GAS5.

In recent years, lncRNAs have been found to play important regulatory roles in gene expression, and abnormal expression of lncRNA is increasingly recognized as a hallmark feature of cancer [40]. Studying these molecules as biomarkers or therapeutic targets will be a promising area for cancer treatment. SNPs represent the most common genetic variants in the human genome. These polymorphisms have a wide distribution and can be found in any region of gene or mRNA. The SNPs that have functional implications on the levels of gene expression are called regulatory SNPs (rSNPs), while those that affect translation, splicing, efficiency to enhance or inhibit the alternative, mRNA stability, and protein function (without altering its structure), they are called structural RNA SNPs (srSNPs) [41]. Nearly, more and more researchers have begun to pay attention to the important role of SNP in cancer. In this article, rs145204276 plays an important role in hepatocarcinoma, colorectal cancer, and gastric cancer tissues, and changes the original tumor suppressor effect of GAS5 (Figure 1). We cannot control the occurrence and changes of SNPs calmly, which makes the treatment difficult, but one day we can overcome it.

**Figure 2:**
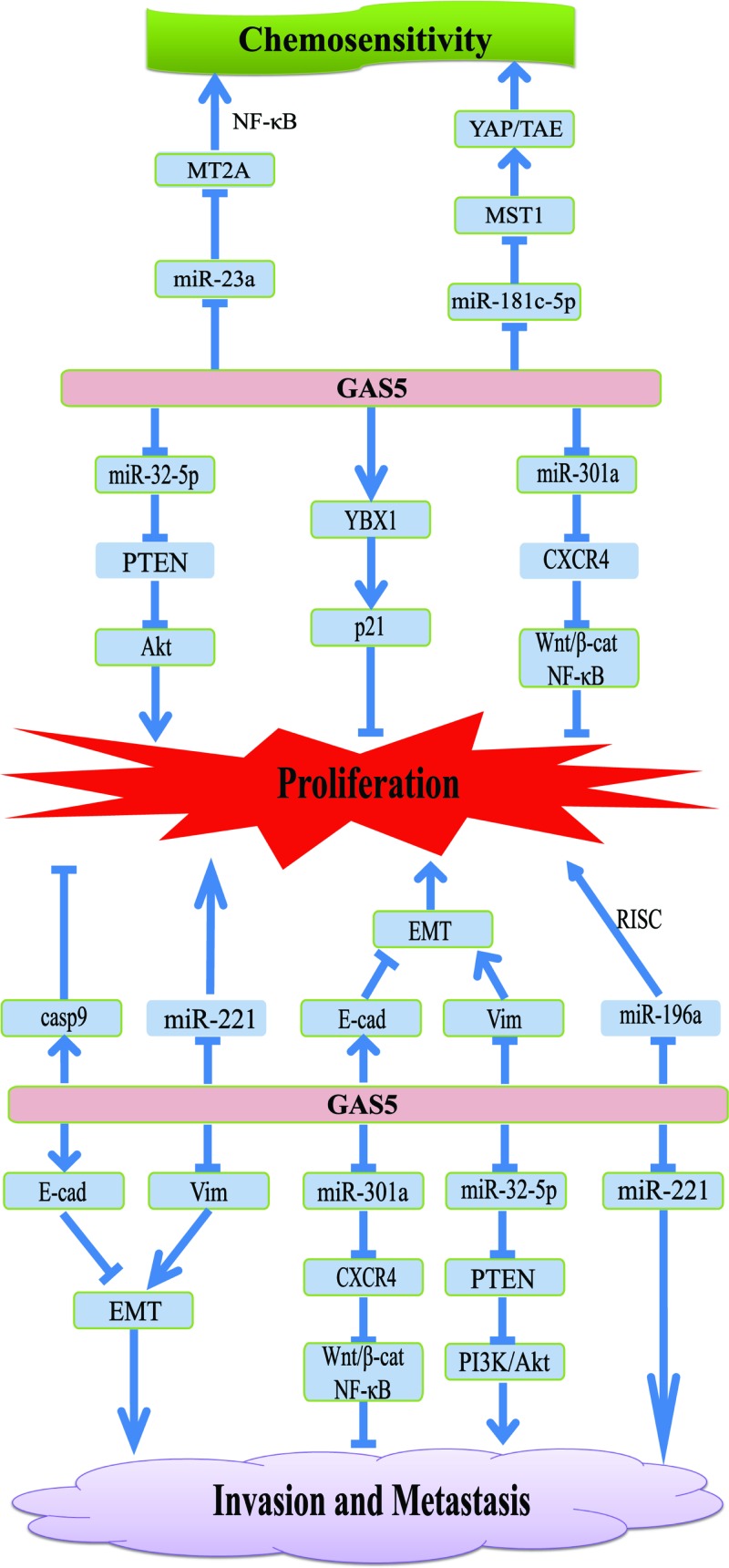
Mechanism and effect of GAS5 on tumor proliferation, metastasis, and chemoresistance The arrow indicates the promotion function and the T type indicates the suppression function.

GAS5 was identified as a tumor suppressor lncRNA in a variety of malignant tumors [42]. GAS5 up-regulation inhibits tumor proliferation, invasion, and migration by regulating related miRNAs, inhibiting EMT processes, activating signaling pathways, and inhibiting cell cycle progression (Figure 2). However, the precise molecular mechanisms upstream and downstream of GAS5 have not yet been fully understood and are yet to be systematically studied. Although most people think that GAS5 is a tumor suppressor lncRNA, there are still some reports that GAS5 has the effect of promoting tumor proliferation. Research report said GAS5 expression was low in liver or spleen but abundant in other adult mouse tissues including brain, heart, thymus, lung, kidney, and testis [43]. This difference may explain the different roles of GAS5 in different tissue carcinogenesis. While, this mechanism is still not clear and needs further analysis and confirmation. The author believes that the relationship between GAS5 and tumor resistance to chemotherapeutic drugs is a very important research direction. For example, GAS5 has been reported to reduce the resistance of pancreatic cancer to gemcitabine and enhance the chemosensitivity of gastric cancer. However, such reports are few and the research depth is not enough. The author believes that the research direction has greater clinical application prospects. It is necessary to systematically study a larger patient cohort to accelerate its clinical application.

## Abbreviations

AFP: α-fetoprotein
Akt: protein kinase B
Casp9: cysteine-containing aspartate-specific proteases 9
E-cad: E-cadherin
EMT: epithelial–mesenchymal transition
ERK: extracellular regulated protein kinase
GRE: glucocorticoid receptor response element
HBsAg: surface antigen of the hepatitis B virus
lncRNA: long non-coding RNA
lncRNA GAS5: lncRNA growth arrest-specific 5
MST1: mammalian sterile 20-like kinase 1
MT2A: metallothionein 2A
NF-kB: nuclear factor kappa-light-chain enhancer of activated B cells
PI3K: phosphatidylinositol 3′-kinase
PTEN: phosphatase and tensin homolog
RISC: RNA-induced silencing complex
rSNP: regulatory single nucleotide polymorphism
SNP: single nucleotide polymorphism
TAZ: transcriptional co-activator with PDZ-binding motif
Vim: Vimentin
YAP: yes-associated protein
YBX1: Y-box binding protein 1
β-cat: β-catenin
